# The effect of alpha lipoic acid ingestion on glucose uptake in healthy males

**DOI:** 10.1186/1550-2783-8-S1-P32

**Published:** 2011-11-07

**Authors:** Iva Mandic, Jason Vescovi, Michael Riddell, Ira Jacobs

**Affiliations:** 1 School of Kinesiology and Health Science, York University, Toronto, ON M3J 1P3, Canada; 2 Faculty of Physical Education & Health, University of Toronto, Toronto, ON M5A 2N4, Canada

## Background

There are reports that indicate dietary alpha lipoic acid (ALA) supplementation enhances glucose uptake. The research was done with animal models and diabetic humans, but the effects of ALA supplementation on glucose uptake in healthy humans are unknown. The present study was designed to test the hypothesis that acute ingestion of ALA would enhance glucose uptake in healthy male subjects.

## Methods

Thirteen healthy, male volunteers (age, 22.2 ± 2.8 years; body mass, 76.5 ± 11.1 kg; mean ± SD) were recruited to participate in a randomized single-blind crossover study. Subjects were administered two fasting oral glucose tolerance tests (OGTT) to clarify if ALA enhanced their glucose uptake. Subsequently, on 2 different occasions with at least one intervening week, subjects cycled at 75% of VO_2max_ for an hour and then completed three to four 5-min bouts at 90% of VO_2max_ with 5 min of active recovery between bouts. Following exercise, subjects were supplemented with either 1g/kg bw of carbohydrate solution, or 1g/kg bw of carbohydrate and 4mg/kg bw of ALA every hour for 4 h post exercise. During this recovery period, venous blood samples were obtained and immediately assayed for plasma glucose concentration using an automated glucose analyzer. Serum insulin values were subsequently assayed using the IMMULITE 2000 immunoassay system. Both absolute concentrations and the areas under the curve for the glucose and insulin concentrations were compared between the ALA and placebo trials.

## Results

Regardless of treatment, the AUC_0-120min_ for glucose (12.7±1.6mmol/L·h^-1^ for placebo; 13.2±1.8 mmol/L·h^-1^ for ALA) and the AUC_0-120min_ for insulin (500±130pmol/L·h^-1^ for placebo; 516±1712 pmol/L·h^-1^ for ALA) remained unchanged during the OGTT (P>0.05). However during the four hours post exercise, there was a main effect for treatment; glucose values were significantly higher in the ALA condition (7.1±1.8mmol/L for ALA vs. 6.5±1.8mmol/L for placebo; P<0.05). Insulin values were also significantly higher at 180 minutes post exercise in the ALA condition (656±359 pmol/L) compared to placebo (472±206 pmol/L; P<0.05).

## Conclusion

In contrast with earlier reports of the effects of ALA in animals and diabetic humans, this study concludes that enhancement of glucose uptake does not occur in healthy males. The ALA treatment interaction causing higher insulin and glucose values during recovery from exhaustive exercise should be further studied.

